# Subject-Independent Emotion Recognition Based on EEG Frequency Band Features and Self-Adaptive Graph Construction

**DOI:** 10.3390/brainsci14030271

**Published:** 2024-03-12

**Authors:** Jinhao Zhang, Yanrong Hao, Xin Wen, Chenchen Zhang, Haojie Deng, Juanjuan Zhao, Rui Cao

**Affiliations:** School of Software, Taiyuan University of Technology, Taiyuan 030024, China; zhangjinhao1261@link.tyut.edu.cn (J.Z.); haoyanrong@tyut.edu.cn (Y.H.); xwen@tyut.edu.cn (X.W.); 2022511314@link.tyut.edu.cn (C.Z.); 2022511305@link.tyut.edu.cn (H.D.); zhaojuanjuan@tyut.edu.cn (J.Z.)

**Keywords:** electroencephalogram, frequency band, subject-independent, adjacency matrix, SEED dataset, deep learning

## Abstract

Emotion is one of the most important higher cognitive functions of the human brain and plays an important role in transaction processing and decisions. In traditional emotion recognition studies, the frequency band features in EEG signals have been shown to have a high correlation with emotion production. However, traditional emotion recognition methods cannot satisfactorily solve the problem of individual differences in subjects and data heterogeneity in EEG, and subject-independent emotion recognition based on EEG signals has attracted extensive attention from researchers. In this paper, we propose a subject-independent emotion recognition model based on adaptive extraction of layer structure based on frequency bands (BFE-Net), which is adaptive in extracting EEG map features through the multi-graphic layer construction module to obtain a frequency band-based multi-graphic layer emotion representation. To evaluate the performance of the model in subject-independent emotion recognition studies, extensive experiments are conducted on two public datasets including SEED and SEED-IV. The experimental results show that in most experimental settings, our model has a more advanced performance than the existing studies of the same type. In addition, the visualization of brain connectivity patterns reveals that some of the findings are consistent with previous neuroscientific validations, further validating the model in subject-independent emotion recognition studies.

## 1. Introduction

Emotion is one of the most important high-level cognitive functions of the human brain, appearing in all aspects of human life. Different emotions can affect the physical health of human beings and, at the same time, emotion also plays an important role in the processing of affairs and rational decision-making [1]. Recognition of emotion is an advanced embodiment of artificial intelligence, and related research has become a hotspot of cross-research in many interdisciplinary fields, such as computer, neuroscience, psychology, brain science, biomedical engineering, and robotics, aiming to analyze, explain, and recognize human emotions and provide results that will promote our understanding of the cognitive mechanism of emotion [2]. The human brain is an extremely complex system in nature, and its complexity is not only manifested in the hundreds of billions of neurons and trillions of connections but also in the diversity of connection patterns, i.e., in the different patterns of connections manifested in cognition, thought, sensation, and behavior. Furthermore, the connection patterns of the brains of different individuals are not the same [3].

With the advantages of noninvasiveness, high temporal resolution, and easy acquisition, EEG signals are widely used in brain science research. In the past decades, many neuropsychological studies have revealed the correlation between cortical regions and human emotions. The different emotions generated in the hypothalamus, amygdala, hippocampus, deep limbic system, and anterior band gyrus are aroused and relayed through the brainstem reticular formation, which is modulated and integrated by the frontal and temporal lobes of the cerebral cortex [4,5,6]. The contribution of EEG signals from different regions of the cerebral lobe is different during emotional cognition [7].

In the field of EEG emotion recognition, various studies have been devoted to exploring effective methods for extracting nonlinear and complex EEG features to recognize emotions. It has been shown that introducing handcrafted feature extraction into classification models can improve emotion recognition performance to some extent. Various hand-crafted features with superior ability to enhance different emotional features were employed. For example, higher-order cross-features [8] and Hjorth features [9], which are time-domain features, are capable of extracting temporal information from a signal. Power Spectral Density (PSD) [10], Wavelet Transform [11,12], and Discrete Wavelet Transform [13], which have the ability to capture local features in the frequency domain, have been widely used in the field of processing EEG emotion recognition. In addition, building brain networks by exploring the relationships among EEG channels has also been used as a feature extraction method. For example, some works have constructed brain networks based on Pearson’s correlation coefficient [14], mutual information [15], etc., which are used in brain network modeling. In addition, many works have used entropy measurements to extract discriminative features by measuring the complexity of EEG signals, such as Shannon entropy (ShEn) [16], sample entropy (SampEn) [17], and differential entropy (DE) [18]. Differential entropy has superior robustness and feature extraction ability and has been widely used in EEG signal analysis, especially in emotion recognition based on EEG signals [19,20]

In terms of EEG feature selection, the more common traditional EEG features are time domain features, frequency domain features, and time–frequency domain features. The main features in the time domain such as statistical features [21], fractal dimension features [22], etc., can be used for emotion characterization. In contrast to time domain features, frequency domain analysis methods can reveal the frequency components of a signal [23]. In the frequency domain, power spectral density [20] and approximate entropy [24] are often used in research. With the deepening of the study, researchers have found that in order to more comprehensively respond to the characteristic information of EEG signals, combining time and frequency domain features to form time–frequency domain features should be used for the comprehensive analysis of EEG signals. For example, wavelet transform entropy [25] and discrete wavelet transform features [26] are widely used in the field of EEG emotion recognition and have achieved good performance. Most of the above traditional EEG features are extracted and studied based on a single channel, and according to previous studies, it has been shown that the huge number of neurons and brain regions in the brain are interconnected to form a complex network [27]. In order to further understand the specific emotional states of the brain and consider the correlations and interactions between channels, the research method of constructing inter-channel adjacency matrices using spatial distances of EEG channels [28] and functional connectivity metrics has also been gradually adopted by most researchers. Functional connectivity metrics are usually selected such as the phase-locked value PLV [29], Pearson correlation coefficient PCC [30], etc. However, in most of the previous studies, the extraction of the adjacency matrix was based on a priori knowledge, which did not fully consider the correlation relationship between channels embedded in the EEG signals. In contrast, the method proposed in this study is more capable of utilizing neural networks to adaptively explore the inter-channel relationships in EEG signals to obtain brain connectivity patterns with more emotional representations.

With the continuous development of artificial intelligence, machine learning and deep learning methods are being gradually applied in the research field of EEG emotion recognition, and the more representative ones mainly include support vector machine SVM [31], CNN [32], RNN [33], and other methods, which have achieved good results. It has been shown that traditional neural networks cannot directly deal with non-Euclidean data. EEG signals are discrete and discontinuous in the spatial domain and thus, it is more favorable to construct the structure of EEG effective graphs based on the knowledge of graph theory and use graph neural networks to deal with the information in the graph domain to better characterize the intrinsic relationship between the channels [20]. A graph neural network is a type of neural network that processes data in the graph domain, such as molecular structures, social networks, and knowledge graphs [34]. A graph convolutional neural network is a neural network with faster localized convolutional operations, where the convolutional layers can be stacked K times to efficiently convolve the K-order neighborhood of a node [35]. Therefore, in the field of EEG emotion recognition, graph representations have also achieved satisfactory performance in processing EEG signals, e.g., a Dynamic Graph Convolutional Neural Network (DGCNN) for emotion recognition was proposed, whose graph structure is defined by a dynamic adjacency matrix reflecting the intrinsic relationship between different EEG electrodes [36]. To capture local and global inter-channel relationships, a regularized graph neural network (RGNN) was proposed, which achieved state-of-the-art performance on the SEED and SEED-IV datasets [37]. Since the attention mechanism [38] was proposed, it has been a focal point in the field of deep learning. Thus, Transformer has been widely used in the fields of translation, imaging, etc. The use of the attention mechanism in Transformer helps to capture the long-term relevance of the data and improves the interpretability. Indeed, it is used in the field of Brain–Computer Interfaces to capture deep features from the EEG signals [39]. Therefore, in this paper, inspired by using graph representations with Transformer in the field of brain science, a new adaptive multi-graph layer research method based on Transformer components is proposed.

The issue of subject independence in the field of EEG emotion recognition research has been of great interest. Subject independence refers to the use of different individuals as subjects in experiments to verify the scalability and robustness of emotion recognition models. EEG signals are usually weak and prone to be adulterated with noise, as well as non-stationary properties, resulting in large differences in EEG signals between subjects, or even between the same subject over longer time spans. In a previous study, a multi-source domain adaptive approach was proposed that considered both domain-invariant and domain-specific features, and a one-to-one domain adaptation method was used to extract domain features to reduce the impact of EEG variability on emotion recognition studies [40]. Therefore, in this study, the subject-independent experimental division method is used as the only index to evaluate the model, aiming to explore the performance of the proposed method in the EEG heterogeneity problem, and the results show that the proposed method achieves more stable performance.

Meanwhile, among the existing research methods for EEG-based emotion recognition, both machine learning and deep learning algorithms have achieved better results. These algorithms are more appropriate for dealing with complex problems by virtue of their strong learning ability and have been introduced into EEG signal-based emotion recognition. However, there are still some shortcomings in the current research including the following:

First, frequency band features should not be neglected because it has been shown that EEG signals of different frequency bands have different relevance in emotion recognition [20]. Second, multichannel EEG signals have structural characteristics of biological topography in non-Euclidean domains [36]. Directly applying deep learning methods to EEG-based feature recognition does not allow for better characterization of emotions, as these methods are designed for computer vision and natural language processing tasks. Previous research methods have manually extracted connectivity metrics between different brain regions through a priori knowledge. However, due to the non-smoothness and specificity of EEG, in the field of emotion recognition research, the use of self-adaptive methods to measure brain connectivity patterns has become a new perspective to explore connectivity patterns, which dynamically determines connectivity metrics between different brain regions from input EEG signals or features and provides better access to the connectivity metrics between different emotional states of the channels and the connectivity between brain regions [41]. In addition, EEG signals vary significantly between individuals, which makes subject-independent emotion recognition studies a challenge.

To solve the above three problems in EEG-based emotion recognition research, we propose a neural network model based on extracted frequency band layer features (BFE-Net), which is a new frequency band-based self-adaptive graph construction emotion recognition model, and fully consider the significance of subject-independent research in our experiments.

(1) In order to research the contribution of EEG frequency bands to emotion recognition, we use DE features extracted from five frequency bands as inputs to the model.
(2) To explore the spatial topological information embedded in EEG signals, we use CNNs and Transformer models to adaptively extract the frequency band graph layer structure.
(3) We use a Graphical Convolutional Neural Network (GCN) to aggregate features to obtain a single-band representation of emotion and recognize emotion by fusing features from the five bands.

## 2. Materials and Methods

This section presents two experimental datasets as well as a detailed description of the proposed method. Finally, based on the selected datasets, the proposed method is subjected to experimentation to explore its performance and relevance in the field of EEG emotion recognition.

### 2.1. Datasets and Preprocessing

In this study, experiments were conducted with the help of two publicly available datasets, including SEED (Synthetic Eyeblink EEG Dataset) and its derivative SEED-IV (Simulated EEG Eyeblink Dataset—Image Version), which are published at https://bcmi.sjtu.edu.cn/~seed/ (accessed on 13 March 2021).

**Figure 1:**
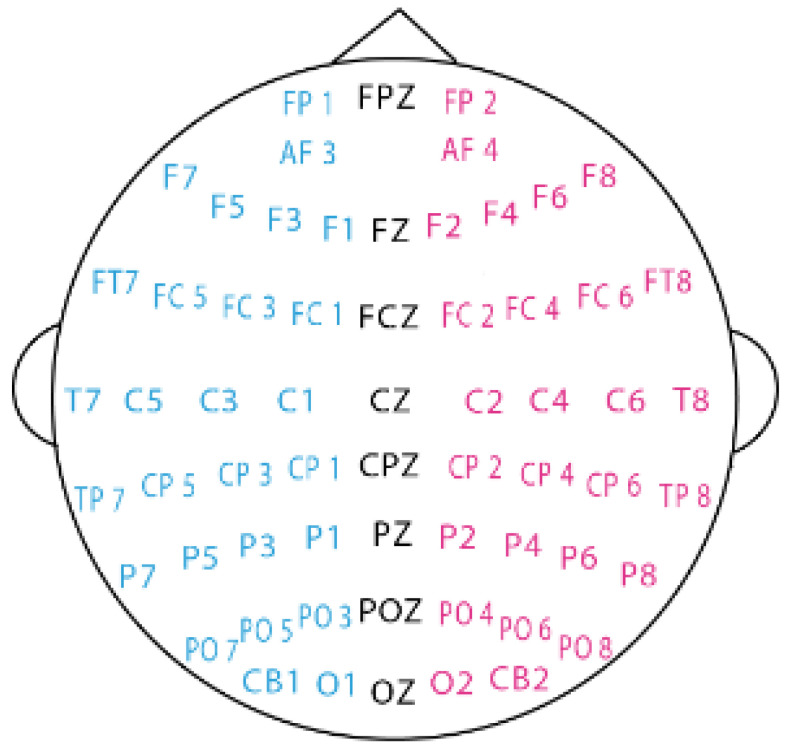
Schematic diagram of the 62 electrodes in the EEG caps used for the SEED and SEED-IV datasets. The figure shows the approximate location of each electrode in the brain.

The SEED dataset contains EEG data from 15 subjects (7 males) recorded in 62 EEG channels using the ESI NeuroScan system, e.g., Figure 1. These data were collected while the participants watched movie videos of three emotion types including negative, neutral, and positive, each lasting approximately 4 min. SEED collected emotional data during three different experimental times, with each subject watching 15 movie videos of different emotional types in each trial. There is a total of 675 EEG samples in the SEED dataset (45 trials × 15 subjects). For each subject, there are 15 samples for negative, neutral, and positive emotion types.

The SEED-IV dataset similarly recorded EEG data in 62 EEG channels from 15 subjects. The acquisition equipment was the same as that used in SEED. These data were collected while participants watched movie videos of four emotion types, including neutral, sad, fearful, and happy, each lasting approximately 2 min. Emotion data were also collected during three different experimental times, with each subject watching 24 movie videos of different emotion types in each experiment. There is a total of 1080 EEG samples in the SEED-IV dataset (72 trials × 15 subjects). For each subject, there are 18 samples in each category, so the number of samples in each category is balanced in both datasets.

During data preprocessing, the EEG data were down-sampled to 200 Hz and filtered using a 0–75 Hz band-pass filter to divide the EEG data into 4 s non-overlapping time windows of data. To make a fair comparison with existing studies, the differential entropy (DE) feature provided by the dataset, smoothed by a linear dynamical system (LDS), was used directly in this experiment. Differential entropy extends the concept of Shannon’s entropy and measures the complexity of continuous random variables, and according to previous studies, DE features are more effective for emotion recognition compared with other features. For each EEG signal per second in each EEG channel, DE features on five frequency bands (delta, theta, alpha, beta, and gamma) were extracted. Therefore, the data format of DE features for each subject in one experiment was the following: 62 × W × 5, where 62 denotes the EEG channel, 5 denotes the five frequency bands mentioned above, and W denotes the number of time windows in each trial, with different trials having different W values because the video durations are not exactly equal in different trials.

In the SEED dataset, W varies from 185 to 265, while in the SEED-IV dataset, W varies from 12 to 64. To standardize the data lengths, the length of the SEED dataset was standardized to 265, the length of the SEED-IV dataset was standardized to 64, and the features with a short temporal window were used with zeros as padding. Therefore, the data format of each feature sample of the SEED dataset was 62 × 265 × 5 and that of the SEED-IV dataset was 62 × 64 × 5.

This study evaluates and compares different emotion recognition models based on the publicly available datasets SEED and SEED-IV.

### 2.2. Proposed Methodology

**Figure 2:**
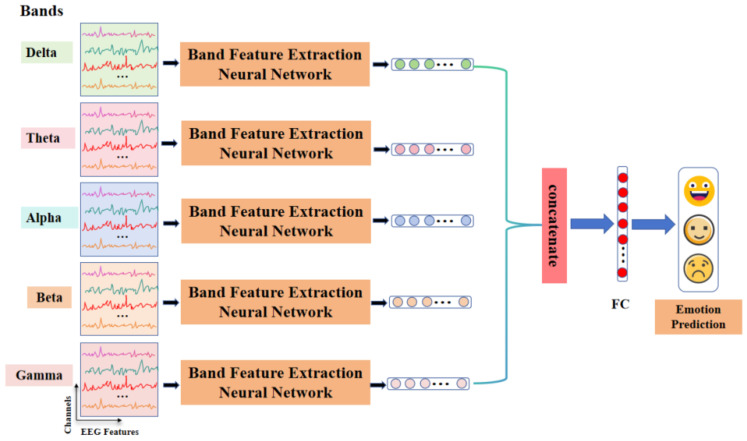
Schematic diagram of the architecture of an emotional network based on EEG frequency bands. The input of the network is the feature of five frequency bands. After learning by the Band Feature Extraction Neural Network, the fused features are used for emotion recognition, and the output of the network is emotion labels.

An overview of the general model proposed in this study is shown in Figure 2. To fully consider the contribution of frequency band information in EEG signals to emotion recognition, a frequency band-based EEG emotion classification network is designed in this study. The model implementation will be publicly available at https://github.com/Doubleb0424/BFEnet (accessed on 2 March 2024). The input of the model is represented as $X\epsilon R^{N\times T\times C}$, where N denotes the number of EEG channels, T denotes the sample length of a single band DE feature, and C Indicates the number of frequency bands characterized by DE, i.e., C=5, where the five bands are delta, theta, alpha, beta, and gamma respectively. After that, it is fed into the frequency band feature extraction network to aggregate the features, and the obtained features are spliced to obtain the full band fusion features. Finally, the emotion recognition is realized after the fully connected layer and SoftMax layer.

**Figure 3:**
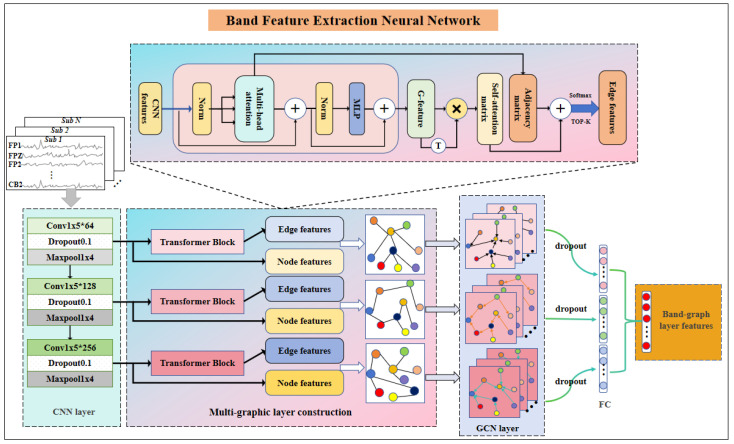
The proposed BFE-Net model, whose inputs are single band features. The model uses a CNN layer and layer construction with a GCN layer to obtain a single-band sentiment representation.

In a single band feature extraction module, a Band Feature Extraction Neural Network (BFE-Net) is proposed, as shown in Figure 3. The BFE-Net consists of three main modules, namely, the convolutional neural network layer (CNN layer), the multi-graphic layer construction module, and the graph convolution and feature fusion layer (GCN layer). The DE features of each frequency band are fed into the BFE-Net, which first goes through the convolutional layer to extract the deep features, is then fed into the multi-graphic layer construction module to construct the graphic layer features, and, finally, is fed into the graph convolutional neural network to aggregate the features and obtain the feature representation of a single band.

#### 2.2.1. CNN Layer

The input DE features are fed into the convolutional layer, which plays an important role in the model. The BFE-Net model can extract EEG features at different levels of abstraction using the convolutional layer. Taking the SEED dataset as an example, its input size is 62 × 265 (number of EEG channels × DE feature dimension), which represents the DE feature data of a single frequency band of a subject. To make the input DE features more capable of characterizing emotion, and considering the small dimension of DE feature data, a CNN is chosen to be used to aggregate the features. Based on the non-Euclidean characteristics of EEG signals and the need to use single-channel features as the node features in the graph structure when constructing the graph structure, as well as to ensure that the features between EEG channels do not mix with each other and then extract the single-channel EEG features, we chose to use a one-dimensional convolutional neural network to extract the single-channel features of the EEG. The module consists of three consecutive convolutional layers, each of which consists of a one-dimensional convolutional kernel, a dropout, and a maximum pooling layer. The convolutional kernel sizes were all selected as 1 × 5, and it was proposed to extract the EEG single-channel features at different abstraction levels with 64,128,256 convolutional kernels in the three convolutional layers, respectively. The dropout was set to 0.1 to prevent the overfitting phenomenon from occurring, while the maximum pooling layer was applied to down-sample the features afterward. In the first and second layers of the network structure, as shown in Figure 3, the outputs of the CNNs are fed into the connected multi-layer construction module and the next layer of CNNs, respectively, aiming to deepen the depth of the network while carrying out deep feature extraction of the original DE features and obtaining the single-channel features with a greater characterization capability.

#### 2.2.2. Multi-Graphic Layer Construction

**Figure 4:**
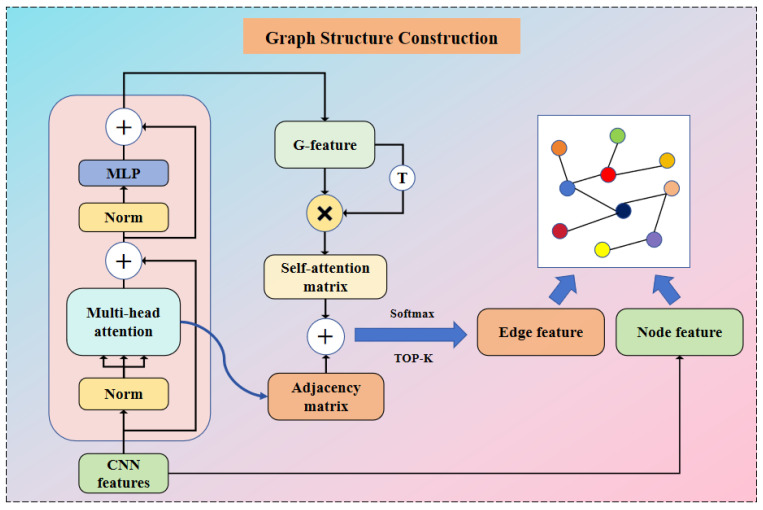
Graph structure construction. A single graphics layer is constructed by using the self-attentive adjacency matrix output of the Transform module as edge features and the output of the CNN as point features. ⊕ represents matrix addition, ⊗ represents matrix multiplication, and T represents matrix transpose.

This module uses the CNN feature as input and proposes a new method of adaptively learning the adjacency matrix (i.e., characterizing the correlations between different EEG channels) using neural networks. It uses the Encoder component in the Transformer model to adaptively extract the adjacency matrix, and then later uses this adjacency matrix with the CNN feature constructed as a graph structure as the layer structure of this network hierarchy. The way the graph is constructed for each layer is shown in Figure 4.

The distribution positions of the electrodes of the EEG cap are defined by a number of standards, such as the International 10/20 System. The distribution positions of the electrodes are fixed and regular, and thus, EEG signals can be considered as classical non-Euclidean structured data, which are well suited for graphical data representation.

In addition, inspired by the successful application of Transformer in the field of NLP, researchers introduced it into the field of computer vision and proposed the VIT-Transformer model, which compensates for the shortcomings of Transformer in processing sequential data and can utilize the mechanism of self-attention to better capture spatial and temporal information. Therefore, in this study, the Encoder part of VIT-Transformer is introduced and improved, which is utilized to adaptively extract the intrinsic correlation relationship between different EEG channels and then obtain the edge features in the graph structure. In each layer of the network structure, the feature data outputted from the CNN are fed into the Encoder. Let the input feature data be $X_{i}$:

(1) $$X_{n1}={LayerNorm}_{1}(X_{i})$$

where LayerNorm representation layer normalization, which is a technique used to normalize each sample in the network in terms of feature dimensions. $X_{n1}$ represents the output data after layer normalization. Then, $X_{n1}$ is sent into the multi-attention module. In this study, an adjacency matrix extraction method based on multi-head attention mechanism is proposed, which is calculated as follows:

(2) $$\left\{\begin{array}{c} Q_{i}=X_{n1}\cdot W_{Q}\\ K_{i}=X_{n1}\cdot W_{K}\\ V_{i}=X_{n1}\cdot W_{V} \end{array}\right.$$

where $W_{Q}$, $W_{K}$, $W_{V}$ is the weight matrix for learning. The query ($Q_{i}$), key ($K_{i}$), and value ($V_{i}$) vectors are split into multiple headers, respectively. In Vit-Transformer, it is usually split into the h attention header, a hyperparameter, which can be derived from previous studies and usually takes values of a constant 8 or 12. In the experiments, a smaller number of attention heads can make the model computationally more efficient, and a larger number of attention heads can provide richer expressive capabilities. In this study, the data volume is small, and to ensure that the proposed method is more practical, the number of model parameters cannot be too large, so it is set to h=8.

For the self-attention sublayer in the Encoder section, for each $X_{i}$, its attention output is calculated as:

(3) $$Attention\left(Q_{i},K_{i},V_{i}\right)=softmax\left(\frac{Q_{i}K_{i}^{T}}{\sqrt{d_{k}}}\right)V_{i}$$

(4) $$A=Dropout\left[softmax\left(\frac{Q_{i}K_{i}^{T}}{\sqrt{d_{k}}}\right)\right]$$

where $\sqrt{d_{k}}$ denotes the scaling factor and $d_{k}$ is the dimension of $Q_{i}$ and $K_{i}$ in the attention header. In this study, the transpose matrix of the query value $Q_{i}$ and the key value $K_{i}$ is subjected to a matrix product operation, which is used to characterize the correlation relationship between the EEG channels embedded in the EEG features after the softmax and Dropout operations. It is represented by using A in Equation (4), i.e., the adjacency matrix in Figure 4.

(5) $$X_{n2}={LayerNorm}_{2}\;\left(X_{i}+MultiHead\left(X_{n1}\right)\right)$$

(6) $$G=MLP\left(X_{n2}\right)+\left(X_{i}+MultiHead\left(X_{n1}\right)\right)$$

where $X_{n2}$ denotes the output of the MLP after performing residual concatenation with normalization operation as in Equation (5). Then, $X_{n2}$ is input to Equation (6), where MLP denotes a feed-forward neural network, which is used to transform the features non-linearly. G  denotes the output of the encoder part of the method, i.e., the G-feature in Figure 4, which contains the global and local feature information in the EEG.

(7) $$S=GG^{T}$$

(8) $$E=softmax\left(A+S\right)$$

The multiplication operation of G with the self-transpose vector is performed to obtain a new matrix S, denoting the self-attention matrix in Figure 4. Then, the self-attention matrix is added with the output adjacency matrix in the multi-head attention, and the softmax activation function is used to obtain the bounded and positive new adjacency matrix, denoted by E in Equation (8), to represent the edge features in the graph structure, i.e., the edge feature in Figure 4. The significance of this is that the EEG features, after being partially learned by Encoder, contain more global and local emotional representations, which are expressed after transposing and multiplying them, and the global and local connections embedded in the EEG channels are better presented. In each network depth, the edge features of inter-channel connections and graph structures are dynamically determined by the corresponding input features.

In general, the computational cost of sparse graphs is much lower than that of complete graphs. In this study, to construct the sparse graph structure, we introduce the top-k technique, i.e., the first k largest weights in the adjacency matrix are retained while the remaining small connection weights are set to zero. The top-k operation is applied as follows:

(9) $$\left\{\begin{array}{l} \;for\;i=1,2,\cdots N\\ \;\;\;index=argtopk\left(E\left[i,:\right]\right)\\ \;\;\;E\left[i,i\bar{ndex}\right]=0 \end{array}\right.$$

where $argtopk\left(\cdot \right)$ is a function to obtain the indexes of the first k largest values of each vector $E\left[i,:\right]$ in the adjacency matrix E. $i\bar{ndex}$ denotes the range of indexes that do not belong to the first k values in $E\left[i,:\right]$. In the generated adjacency matrix, after passing through the top-k technique, only the first k maxima in each row vector of the adjacency matrix are retained, while the remaining values are assigned as 0. In fact, the top-k technique can be considered as an improved maximum pooling layer.

#### 2.2.3. Graph Convolution and Feature Fusion

Different graph structures are dynamically constructed by corresponding input EEG features using a multi-graphic layer construction. The newly constructed graphs can then be processed by graph convolutional layers to extract local and global connectivity features for emotion recognition. Since the constructed graph layer features are different due to different CNN features at different abstraction levels, a graph convolutional neural network is chosen to process the graph features of the three layers.

(10) $$G=\left(V,\varepsilon \right)$$

(11) $$V=\left\{V_{i}│i=1,2,\cdots ,N\right\}$$

(12) $$\varepsilon =\left\{\varepsilon_{ij}│V_{i},\;V_{j}\epsilon V\right\}$$

(13) $$E=\left\{e_{ij}\right\}$$

Single-channel EEG signals collected by the EEG cap can be considered as nodes of a graph. Therefore, we consider a multichannel EEG signal as a graph structure. G denotes the graph, V denotes the set of vertices in graph G, and ε denotes the set of edges in graph G. N is the number of EEG channels in the EEG signal. In the graph structure representation, node $V_{i}$ is typically used to represent a single-channel EEG signal, while edge $\varepsilon_{ij}$ denotes the connectivity between node $V_{i}$ and $V_{j}$. E denotes the adjacency matrix of graph G. $e_{ij}$ denotes the strength of the associated connectivity between nodes $V_{i}$ and $V_{j}$. The set of edges in the multi-graphic layer construction obtained through the above modeling is determined by the dynamic determination of the multi-graphic layer construction. The single-channel EEG features are determined using the output features of the CNN and applying the top-k technique on the edge set construction in order to generate the sparse multi-graphic layer construction.

For the graph structure G, the core of the graph convolutional neural network lies in the message-passing operation through the adjacency matrix E, which is computed as follows:

(14) $$\hat{E}=D^{-\frac{1}{2}}ED^{-\frac{1}{2}}$$

(15) $$\hat{L}=I-\hat{E}$$

(16) $$\hat{L}=U\Lambda U^{T}$$

The first step is to compute the degree matrix D, whose diagonal element $D_{ii}$ denotes the degree of node i, and obtain the normalized adjacency matrix $\hat{E}$. $\hat{L}$ denotes the normalized Laplacian matrix, and eigen-decomposition of $\hat{L}$ yields the eigenvector matrix U and the diagonal matrix Λ. Then, the graph convolution kernel is defined as Θ. The spectral graph convolution is computed as follows:

(17) $$H^{\prime }=\sigma \left({\hat{D}}^{-\frac{1}{2}}\hat{E}{\hat{D}}^{-\frac{1}{2}}X\Theta \right)$$

where $H^{\prime }$ is the node feature representation of the output, σ denotes the Relu activation function, and $\hat{D}$ is the diagonal matrix with diagonal elements $D_{ii}=\sum_{j}{\hat{A}}_{ij}$. The core idea is to utilize the spectral information of the graph to perform a convolution operation by feature decomposition of the Laplace matrix. This enables spectral graph convolution to perform effective feature propagation to nodes while preserving the graph structure.

The output of the graph convolution layer is expanded and concatenated into feature vectors in each BFE-Net to characterize the feature output of that frequency band. The feature vectors of the five frequency bands are concatenated and fed into a fully connected layer with a SoftMax activation function to predict emotional categories. The band emotional recognition model in this study can be trained by minimizing the cross-entropy error between its predicted and true values.

### 2.3. Experimental Design

The SEED and SEED-IV benchmark datasets were experimented with the constructed network model. The following sections describe how the dataset was divided and how the network model parameters were set during the experiment.

#### 2.3.1. Dataset Partitioning Methods

To assess the robustness and generalizability of the model, all data from the three experiments of all subjects were selected in this study, and the leave-one-subject-out (LOSO) cross-validation approach for 15 subjects was chosen to divide the data. Specifically, in each experiment, the DE features of 14 subjects in SEED/SEED-IV were used as the training dataset, and the DE features of the remaining 1 subject were used as the testing dataset. For SEED, the number of samples in the training dataset was 630 (45 trials × 14 subjects), and the number of samples in the test dataset was 45 (45 trials × 1 subject); for SEED-IV, the number of samples in the training set was 720, and the number of samples in the test set was 360. The features of each subject were subtracted from its mean and divided by the standard deviation to achieve data normalization.

#### 2.3.2. Network Parameter Settings

For the hyperparameters of the model in all the experiments, based on several experimental evaluations, the number of convolutional layers was finally chosen as 3, the dropout of the output fully connected layer as 0.1, the batch size as 64, and the epoch as 100. The value of the hyperparameter k in the adjacency matrix extraction was taken as 10. We used Adam to optimize the model parameters using gradient descent. The average area under the curve (AUC) of the model was monitored from all emotional categories by applying a random dropout operation with a dropout of 0.1 during training. If the average AUC reached 0.99 during the training process, the training process was stopped, and the last saved model weights were used to categorize the subjects for emotion recognition. For SEED and SEED-IV with 15 subjects, each round of experiments was conducted in 15 sessions, and the average validation accuracy was considered as the final performance of the model so that it could be compared and evaluated with other emotion recognition studies.

## 3. Results

This section focuses on the metric performance of the frequency band and self-adaptive graph convolution-based BFE-Net proposed in this paper on the SEED and SEED-IV. It discusses the extent to which the network hierarchy and parameter tuning affect the results, as well as the ability to discriminate among different emotional categories.

### 3.1. Comparison Experiments

To further evaluate the overall performance of the BFE-Net model, we conducted a series of experiments on the public datasets SEED and SEED-IV. Listed below are the prior research methods with high relevance that focused on the effect of segmentation bands on emotion categorization using DE features and the LOSO strategy, the results of which are shown in Table 1. Bold values in the table indicate the optimal values among all methods. In the single-band experiments, the BFE-Net proposed in this study obtained higher accuracy in all bands compared with the other methods with the same feature inputs, and it also obtained lower standard deviations in the remaining four bands compared with the other methods, except for the theta band.

In the experimental results for the full frequency band, BFE-Net obtained an average accuracy of 92.29% in SEED and achieved an average accuracy of 79.81% in SEED-IV, where both values were higher than the other methods using the same feature input. Meanwhile, BFE-Net obtained 4.65% and 4.11% standard deviation in experiments with different subjects, which were lower than the other research methods in the table. This indicates that the BFE-Net proposed in this study has a more stable performance in subject-independent emotion recognition studies.

### 3.2. Analysis of Model Parameters

This section focuses on exploring the effects of the hierarchy and K-value selection in the model on the performance of emotion recognition. Table 2 demonstrates the effect of network depth on the performance of the BFE-Net model in full-band feature input (i.e., Figure 3), where one-layer, two-layer, and three-layer represent the number of network down-sampling layers, respectively. From the results in the table, it can be found that BFE-Net achieves an average accuracy of 85.40%, 88.53%, and 92.29% on SEED, and 73.98%, 75.09%, and 79.81% on SEED-IV, respectively. As the network level deepens, the input features are better learned by the model, resulting in more comprehensive and accurate emotional features, with better performance achieved in subject-independent experiments, but at the same time, the resources consumed by the model also increase. Therefore, the results reported in Table 1 are the performance of the selected three layers.

In the multi-graphic layer construction module, this study uses the Encoder component of Transformer to dynamically obtain an adjacency matrix suitable for the EEG signals of different subjects. After TOP-K filtering, sparse adjacency matrices are obtained so as to construct the corresponding graph structures for obtaining affective representations independently from other subjects. As shown in Table 3, four different K-value sizes, i.e., $\left(K=5,10,15,20\right)$, were selected for this experiment to discuss the effect of the K-value on the performance of emotion recognition, and the inputs to the same model were full-band features. From the table, it can be seen that different classification accuracies are achieved as the value of k is varied, and the best performance of the model is achieved when K=10.

This is due to the fact that both SEED and SEED-IV used in this study have 62 EEG channels, and in the adjacency matrix, selecting a smaller K-value will discard more feature information, which makes the model’s ability to learn to generalize decrease. On the contrary, when a larger K-value is selected, the adjacency matrix automatically acquired by the model will contain more emotional brain connectivity patterns, resulting in an increase in accuracy. However, at the same time, as the K-value increases, the number of model parameters becomes larger, the feature information of the adjacency matrix becomes redundant, and the effective features are less likely to be captured by the model, which reduces the recognition performance of the model. Therefore, the results reported in Table 1 are the performance at the time K=10 was chosen.

### 3.3. Ablation Experiments

To verify the validity of the important modules in the BFE-Net model, we conducted three ablation experiments on the two datasets used for the experiments, as shown in Table 4 for each of the experiments. Specifically, we completed the following:

(1) The validity of the self-adaptive graph structure was verified by constructing the graph structure using the three-dimensional spatial coordinate distances of the EEG channels as the adjacency matrix, which is denoted by w/Distance in the table.
(2) The validity of the adaptive graph structure was verified by constructing a graph structure using the phase-locked value PLV from the functional connectivity metrics as an adjacency matrix, which is denoted by w/PLV in the table.
(3) The validity of the self-attention matrix in the multi-graphic layer construction module was verified, which is denoted by w/o Self-Matrix in the table.

From Table 4, we can see that if the spatial distance is used as the adjacency matrix, the average accuracy of the model decreases by 11.77% in SEED and 14.5% in SEED-IV; when PLV is used as the adjacency matrix, the average accuracy of the model decreases by 4.73% in SEED and 8.82% in SEED-IV; and when the self-attention matrix is removed, the average accuracy of the model decreases by 6.14% in SEED and 4.65% in SEED-IV. The results show that the method of extracting the adjacency matrix adaptively in BFE-Net is superior to the method of extracting the adjacency matrix using spatial distances and functional connectivity. In addition, it is able to effectively capture the specific emotional features of different subjects, thus improving the performance of the model. The introduction of the self-attention matrix can effectively enhance the EEG inter-channel connectivity features of the self-adaptive adjacency matrix.

### 3.4. Confusion Matrix

**Figure 5:**
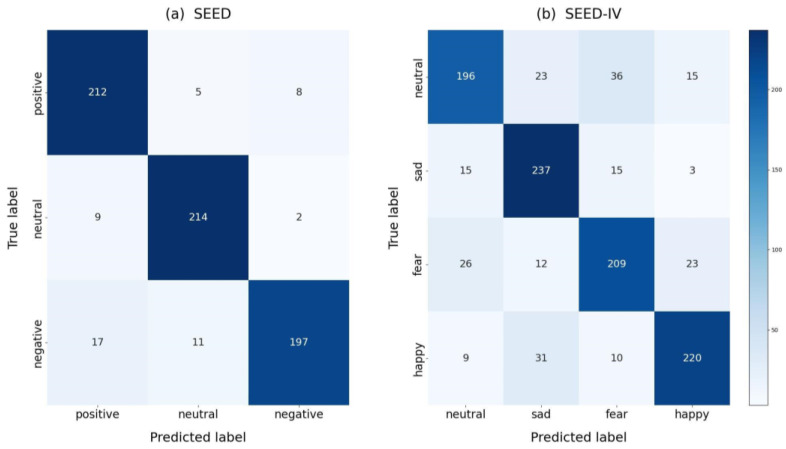
Confusion matrices for the SEED and SEED-IV datasets, denoted by (**a**,**b**). The horizontal coordinates in the figure indicate the predicted labels and the vertical coordinates indicate the true labels. The numbers are used to indicate the number of predictions obtained from the experiments.

To further validate the performance of the BFE-Net model, the confusion matrix based on subject-independent experiments is shown in Figure 5. The horizontal axis represents the predicted labels of the model, and the vertical axis represents the ground truth labels. In the SEED dataset, the labels of the three categories are positive, neutral, and negative from left to right and top to bottom, and neutral, sad, fear, and happy in the SEED-IV, respectively. The BFE-Net model shows better performance in identifying negative, neutral, and positive emotions in SEED in terms of the classification results. The model recognition results were 87%, 95%, and 94%, respectively.

### 3.5. Visualization

**Figure 6:**
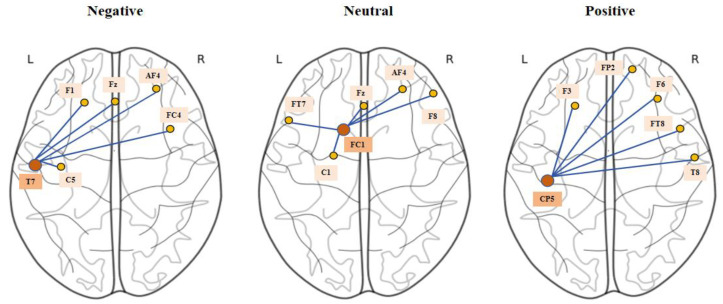
The SEED dataset used as an example to visualize the EEG channels’ activation of BFE-Net under three emotion labels. The yellow dots in the figure represent the EEG channels and the blue lines represent the connections between the channels.

To analyze the inter-channel relationships learned by the proposed model, we choose SEED to visualize the adjacency matrix generated adaptively by the model, as shown in Figure 6. To explore how brain regions are connected and activated under different emotional states, firstly, the single-band adjacency matrix is subjected to an average normalization operation, and then the diagonal elements in the adjacency matrix generated by the three emotions are extracted and converted into a topographic map of the brain.

## 4. Discussion

To further analyze the validity and feasibility of the methodology proposed in this paper, the experimental results are discussed in detail in this section.

We conducted a series of experiments on two datasets, as shown in Table 1. Most of these methods focused on using DE features and frequency band features. In addition, all of them used subject-independent data partitioning to further discuss the feasibility of the proposed methods. BFE-Net obtained higher accuracy with lower standard deviation compared with the same type of studies, which indicates the stability and validity of the proposed method in subject-independent EEG-based emotion recognition. We analyzed in detail the differences between BFE-Net and the existing studies as follows:

(1) SVM [37]: Support vector machine, abbreviated as SVM, is a classical supervised machine learning algorithm. It exhibits many unique advantages in solving small samples and nonlinear and high-dimensional pattern recognition.
(2) SA [42]: A new domain adaptive algorithm. The source and target domains are represented by subspaces described by feature vectors.
(3) DGCNN [36]: Multi-channel EEG-based emotion classification method based on DGCNNs that initializes the adjacency matrix and trains the adjacency matrix dynamically through backpropagation.
(4) TANN [43]: A transferable attention neural network for EEG emotion recognition, which learns the emotional discriminative information by highlighting the transferable EEG brain region data and samples adaptively through local and global attention mechanisms.
(5) BIDANN [44]: A neural network that maps left hemisphere and right hemisphere EEG data into discriminative feature spaces separately, and the feature data are later categorized.
(6) BIDANN-S [45]: A deep learning method for EEG-based emotion classification that uses raw EEG features extracted from each cerebral hemisphere to extract discretized deep features and a domain discriminator to mitigate domain differences between source and target domains.
(7) BIHDM [46]: A bi-hemispheric discrepancy model that learns asymmetrical differences between two hemispheres using four recurrent neural networks to capture information from EEG electrodes in each hemisphere from horizontal and vertical streams.
(8) RGNN [37]: A regularized GNN, which mainly contains node-domain adversarial training and emotion-aware distribution algorithms to achieve emotion recognition
(9) SOGNN [47]: A Self-Organizing GNN for EEG Cross-Subject Emotion Classification, which builds graph structures based on input EEG features self-using and uses GNN learning features for emotion recognition.

The above methods involve algorithms such as machine learning, CNN, GNN, RNN, and domain adaptation, which are used to research subject-independent EEG emotion recognition from different perspectives. In previous machine learning-based studies, such as SVM, feature extraction of EEG was performed based on a priori knowledge. The feature information embedded in the raw EEG data was discarded. This challenge can be solved by utilizing data-driven methods, which use neural networks to learn features for better recognition performance. In previous studies based on domain adaptation, such as SA, the feature space of source and target domains was utilized for emotion recognition, but the spatial structure information embedded in EEG could not be well expressed. BIDANN and BIDANN-S, mentioned in the above methods, integrate the characteristics of the brain’s left and right hemispheric differences with domain adaptation for research. In addition to the above two methods, BIHDM can also effectively assign the contribution of the brain’s left and right hemisphere lateralization to emotion recognition. Brain networks represent the complex connection patterns of the brain, and the introduction of GNN can effectively learn the complex spatial topology in EEG, such as DGCNN, RGNN, and SOGNN. Different graph structures can be constructed by using different adjacency matrices and EEG features. Constructing the graph structure based on a priori knowledge has some limitations and cannot correctly simulate the dynamic EEG of different subjects in different emotional states. DGCNN can dynamically adjust the adjacency matrix, and SOGNN can adaptively extract the adjacency matrix based on different feature inputs. These two methods can dynamically build the graph structure according to the EEG of different subjects. In addition, TANN introduces the attention mechanism and combines brain regions for emotion recognition.

The BFE-Net model proposed in this paper fully considers the advantages and disadvantages of the above methods. BFE-Net takes the frequency band features of EEG as model inputs and utilizes a neural network to fully learn the features. A one-dimensional convolutional kernel is used in the CNN layer to aggregate the EEG single-channel features, which prevents the features of certain channels from being neglected and more realistically restores the EEG generation process. The Transformer model is introduced to adaptively acquire the adjacency matrix, and its output self-attention matrix is added to generate the self-attention adjacency matrix. Because the Transformer model is suitable for processing time series data, the temporal characteristics of the EEG obtain a better representation, and the adjacency matrix contains global information, as shown in the third row of Table 4. In the multi-graphic layer construction module, three graph layers with different network depths are constructed. As the network layers deepen, the graph structure of each layer is dynamically constructed and independent of each other. After feeding the graph structure into the GNN for further aggregation and learning, comprehensive and accurate sentiment features for a single frequency band can be obtained, as shown in Table 2. Finally, the five single-band features are fused for emotion recognition. BFE-Net can construct multi-graphical layers of emotion features independent of each other based on feature inputs from different subjects, which makes it reliable in subject-independent EEG-based emotion recognition.

In the single-band experimental results in Table 1, we compare the performance of each model in different frequency bands. In general, most models, including ours, achieve better recognition performance in the beta and gamma bands than the delta, theta, and alpha bands. SOGNN achieves the best performance in the theta band, followed by the beta and gamma bands. Because the higher frequency bands of EEG signals have better emotional capabilities, the beta and gamma bands obtain a more reliable accuracy, thus improving the accuracy, which is similar to the results of previous studies [37,42,45,47].

In Figure 5, we find that neutral and positive emotions are more easily recognized in the SEED dataset, and negative emotions are more easily recognized as positive emotions. This is because positive and negative emotions are more likely to cause large fluctuations in EEGs, while neutral emotions cause smoother EEGs. In the SEED-IV dataset, sad, happy, and fear emotions are more easily recognized, and neutral emotions are the worst recognized. This is because neutral emotions produce smoother EEGs than the other three emotions, resulting in lower recognition accuracy.

In Figure 6, we plot the pattern of EEG channel connectivity of BFE-Net under different emotions. According to the topographic map, we derive three electrode locations with maximum weights, namely, T7, FC1, and CP5, and show the five electrodes with maximum weights connected to these three channels. In the three emotional states, the channels with the largest weights have a larger weight between the channels with the largest weights and the EEG channels that are mostly from the prefrontal lobe of the brain, i.e., the pattern of brain region connections is mostly concentrated in the prefrontal lobe region of the brain. According to previous studies [48], the activation of frontal lobe regions of the brain is associated with positive and negative emotions. Also, the activation patterns between the left and right hemispheres of the brain are more pronounced according to the planar presentation, and it has been shown in previous studies that the asymmetry of the activity of the EEG between the left and right hemispheres is crucial for emotion recognition [37]. In addition, asymmetric frontal and parietal regional connectivity patterns may reflect the process of emotional change [49]. As demonstrated by previous studies, our findings are consistent with the existing findings.

## 5. Conclusions

In this paper, a new subject-independent emotion recognition model named BFE-Net is proposed. The following are the main contributions of this paper:

(1) A new neural network-based emotion recognition method for EEG frequency bands (BFE-Net) is proposed, which constructs EEG multi-graphic layer emotion features by adaptively acquiring the adjacency matrix.
(2) Based on the frequency band features of EEG, BFE-Net investigates the effect of different frequency bands on emotion recognition. In the process of single-band feature extraction, a new Transformer Encoder-based adaptive extraction of adjacency matrix is proposed to extract the multilayer structure used to characterize emotion according to the network depth. It can also be used to explore emotional activities among different functional brain regions.
(3) BFE-Net obtains optimal performance in subject-independent emotion recognition, with an average accuracy of 92.29% in SEED, as well as 79.81% in SEED-IV. Extensive ablation and comparison experiments are also conducted to validate the performance of the proposed BFE-Net.

Based on the experimental results we can see that BFE-Net achieves advanced performance on two publicly available EEG emotion datasets under the same experimental setup. This indicates that the EEG frequency band features with multi-graph layer map structure are effective in subject-independent emotion recognition. BFE-Net analyzes the EEG features of different subjects independently, which effectively reduces the effect of EEG data heterogeneity on subject-independent emotion recognition. We hope to provide new perspectives for other EEG-based studies.

Admittedly, there are some limitations in this study. The validity of EEG time–frequency domain features was demonstrated in previous studies based on EEG frequency bands. Spectral analysis and wavelet transform can convert EEG to graphs for processing [50]. Wavelet transform can detect and analyze time-varying signals [51]. Fast Fourier Transform (FFT) can convert temporal signals from the time domain to the frequency domain [52]. It has been suggested that empirical mode decomposition (EMD) can decompose an EEG into several intrinsic mode functions (IMFs), which can later be used to compute features using autoregressive models for emotion recognition [53]. Furthermore, in terms of neural network architectures, previous studies have shown that domain adaptation can effectively reduce the effect of variability in EEG data [37,45,54]. Multimodal data have also been applied in previous studies. The fusion of eye movement data with EEG data can improve the performance of emotion recognition [55].

In our future research, we will complete the following:

(1) Explore more effective EEG features and introduce EEG spectral analysis, wavelet transform, empirical pattern decomposition, and other feature analysis methods. Build complex brain networks to study brain-specific connectivity patterns in emotional states and effectively decode high-level cognitive behaviors.
(2) Explore other neural network architectures such as recurrent networks using LSTM cells and autoencoders in the study of EEG frequency bands and brain networks.
(3) Improve the performance of emotion recognition and further explore human physiological states in emotional states. Integrate multimodal physiological data such as eye movement data, EMG signals, and facial expressions into emotion recognition research.

## Figures and Tables

**Table 1:** Subject-independent emotion recognition accuracy (mean/standard deviation) in the SEED and SEED-IV datasets.

	Bands	SEED						SEED-IV
Model		Delta	Theta	Alpha	Beta	Gamma	All Bands	All Bands
SVM [37]		43.06/08.27	40.07/06.50	43.97/10.89	48.63/10.29	51.59/11.83	56.73/16.29	37.99/12.52
SA [42]		53.23/07.47	50.60/08.31	55.06/10.60	56.72/10.78	64.47/14.96	69.00/10.89	64.44/09.46
DGCNN [36]		49.79/10.94	46.36/12.06	48.29/12.28	56.15/14.01	54.87/17.53	79.95/09.02	-
TANN [43]		-	-	-	-	-	84.41/08.75	68.00/08.35
BiDANN [44]		-	-	-	-	-	83.28/09.60	-
BiDANN-S [45]		63.01/07.49	63.22/07.52	63.50/09.50	73.59/09.12	73.72/08.67	84.14/06.87	65.59/10.39
BiHDM [46]		-	-	-	-	-	85.40/07.53	69.03/08.66
RGNN [37]		64.88/06.87	60.69/**05.79**	60.84/07.57	74.96/08.94	77.50/08.10	85.30/06.72	73.84/08.02
SOGNN [47]		70.37/07.68	76.00/06.92	66.22/11.52	72.54/08.97	71.70/08.03	86.81/05.79	75.27/08.19
BFE-Net (ours)		**84.14/05.43**	**86.66**/06.88	**81.48/04.91**	**89.18/05.84**	**88.00/06.68**	**92.29/04.65**	**79.81/04.11**

Note: - indicates that this item was not covered in the study. Bolded numbers indicate the highest accuracy with the lowest standard deviation.

**Table 2:** Impact of network depth on the model performance of BFE-Net (i.e., mean/standard deviation). N−layer represents the number of network layers in the BFE-Net, N=1,2,3.

	Dataset	SEED	SEED-IV
Layers			
One-layer		85.40/05.18	73.98/05.42
Two-layer		88.53/05.01	75.09/04.95
Three-layer		**92.29/04.65**	**79.81/04.11**

Note: Bolded numbers indicate the highest accuracy with the lowest standard deviation.

**Table 3:** Effect of K-value size on the model performance of BFE-Net (i.e., mean/standard deviation).

	Dataset	SEED	SEED-IV
K			
K = 5		90.51/04.40	75.18/05.43
K = 10		**92.29/04.65**	**79.81**/04.11
K = 15		91.11/05.79	74.16/**03.71**
K = 20		89.92/05.18	75.00/04.75

Note: Bolded numbers indicate the highest accuracy with the lowest standard deviation.

**Table 4:** Ablation experiments.

	Dataset	SEED	SEED-IV
Model			
BFE-Net		**92.29/04.65**	**79.81/04.11**
w/Distance		80.52/12.35	65.31/09.23
w/PLV		87.56/10.89	70.99/11.78
w/o Self-Matrix		86.15/09.78	75.16/08.65

Note: Bolded numbers indicate the highest accuracy with the lowest standard deviation.

## Data Availability

The open-access datasets SEED and SEED-IV were used in our study. The links are as follows: SEED: https://bcmi.sjtu.edu.cn/~seed/seed.html (accessed on 13 March 2021). SEED-IV: https://bcmi.sjtu.edu.cn/~seed/seed-iv.html (accessed on 13 March 2021).
